# Measuring vibrations on a biofidelic brain using ferroelectret nanogenerator

**DOI:** 10.1038/s41598-023-35782-5

**Published:** 2023-06-02

**Authors:** Henry Dsouza, Bianca M. Dávila-Montero, Ian Gonzalez Afanador, Gerardo Morales Torres, Yunqi Cao, Ricardo Mejia-Alvarez, Nelson Sepúlveda

**Affiliations:** 1 Electrical and computer engineering, Michigan State University, 428 S Shaw Lane, East Lansing, MI 48824 USA; 2 Mechanical Engineering, Michigan State University, East Lansing, MI 48824 United States; 3 State Key Laboratory of Industrial Control Technology, College of Control Science and Engineering, Zhejiang University, Hangzhou, 310027 Zhejiang China

**Keywords:** Health care, Energy science and technology, Engineering, Materials science

## Abstract

Our knowledge of traumatic brain injury has been fast growing with the emergence of new markers pointing to various neurological changes that the brain undergoes during an impact or any other form of concussive event. In this work, we study the modality of deformations on a biofidelic brain system when subject to blunt impacts, highlighting the importance of the time-dependent behavior of the resulting waves propagating through the brain. This study is carried out using two different approaches involving optical (Particle Image Velocimetry) and mechanical (flexible sensors) in the biofidelic brain. Results show that the system has a natural mechanical frequency of $\sim$ 25 oscillations per second, which was confirmed by both methods, showing a positive correlation with one another. The consistency of these results with previously reported brain pathology validates the use of either technique, and establishes a new, simpler mechanism to study brain vibrations by using flexible piezoelectric patches. The visco-elastic nature of the biofidelic brain is validated by observing the the relationship between both methods at two different time intervals, by using the information of the strain and stress inside the brain from the Particle Image Velocimetry and flexible sensor, respectively. A non-linear stress-strain relationship was observed and justified to support the same.

## Introduction

**Figure 1:**
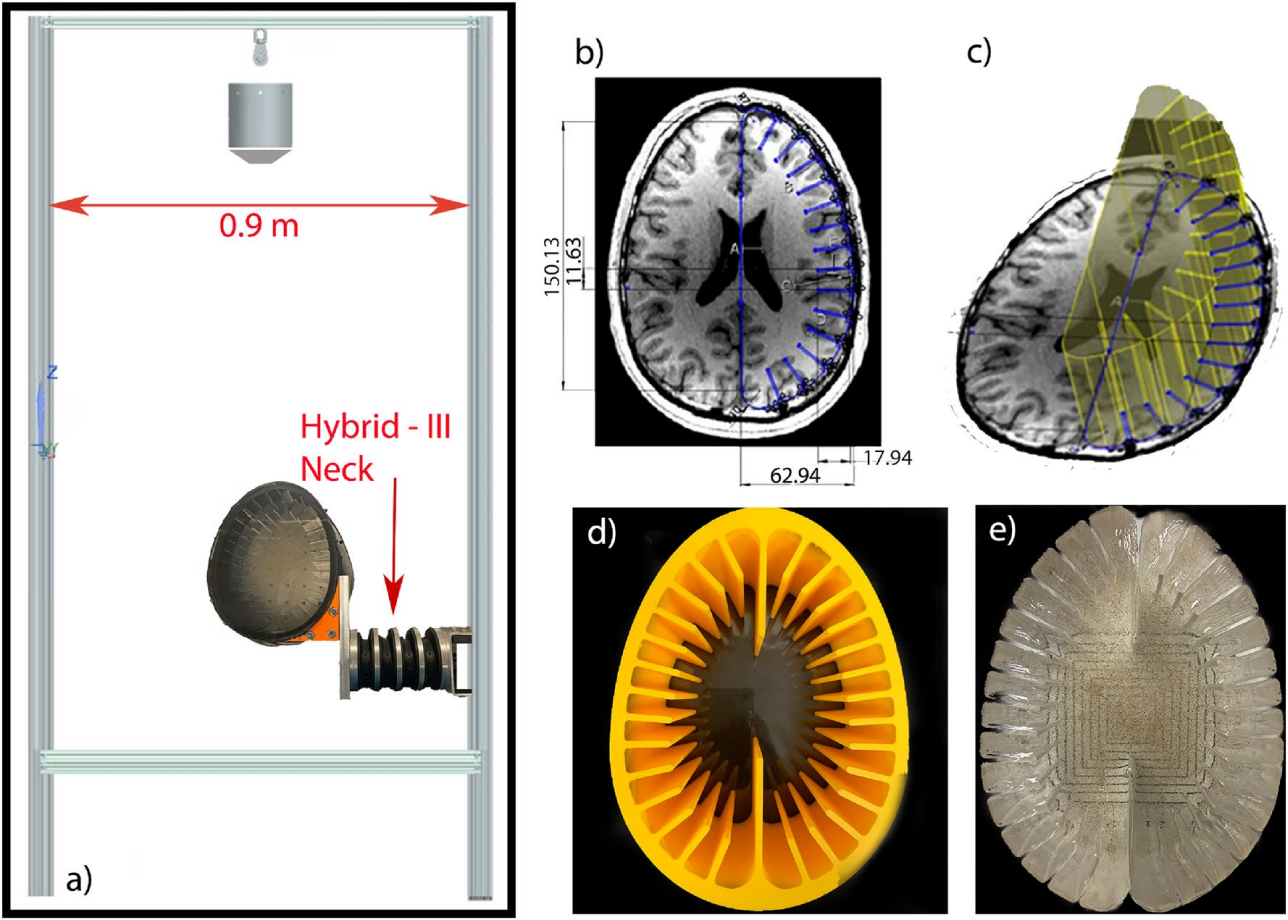
(**a**) Drop tower along with the placement of phantom and neck arrangement. (**b**) MRI from a 35-year-old healthy male and (**c**) extruded section for computer design. Adapted from^18^. (**d**) Mold used to create the brain phantom. (**e**) Brain phantom with embedded finite sand particles.

Traumatic brain injury (TBI) has been one of the major causes of death or disability around the world^1^. TBI incidence in high school football players can be twice as high due to under-reporting because of lack of awareness or a desire to keep playing^2^. Even a milder form of TBI (also known as a concussion), has been recognized as a serious health concern due to their long-term effects^3^, and their link to chronic traumatic encephalopathy (CTE), Alzheimer’s and Parkinson’s disease^4^. This has created a pressing need to better understand and prevent this type of injuries. The Center of Diseases Control and Prevention (CDC)^5^, defines a concussion as *a type of traumatic brain injury (or TBI) caused by a bump, blow, or jolt to the head or by a hit to the body that causes the head and brain to move rapidly back and forth.* Even low magnitude impacts could cause severe brain damage, if the wave propagating through the brain has frequency components within the 20 and 40 Hz range^6^. Thus, it is important to understand the implications of the magnitude of the impact, as well as its time-dependent behavior –i.e. the frequency components of the pressure waves generated by the impact. The brain can be seen as a viscoelastic medium with complex and intricate geometry. An impact to the skull creates traveling waves that propagate at different frequencies and different speeds since the composition is non-homogeneous. This can create localized and time-dependent strain concentrations at certain regions in the brain. Thus, understanding of the temporal dynamics of the brain upon impact is vital for determining the severity of a collision, and its long-lasting consequences. To this end, modeling of the brain has been researched since the 1940’s when Holbourn proposed that the brain can be modeled as a mechanical system with input in the form of head motion and brain displacement as an output^7^. He also states that by knowing the physical properties of the brain the behaviour after a blow can be studied using Newton’s laws of motion. Since then, brain injuries have been characterized by the kinematic signatures of the head, such as the work done by Ommaya and Gennarelli which indicates that brain injury is proportional to peak acceleration and the duration of the peak^8^. This lead to development of metrics such as Wayne State Tolerance Curve (WSTC)^9^, Gadd Severity Index (GSI)^10^ and Head Injury Criteria (HIC)^11^. Recent advancements in imaging techniques such as diffusion tensor imaging (DTI) have shown that there are changes to the white matter in the brain even in the case of repetitive smaller (i.e. not concussive) impacts^12^. These changes are shown to be the result of excessive axons stretching which in-turn damages them^13^. There is also evidence to suggest that strain in deep brain regions with a high density of axon fibers correlate strongly with cognitive impairment or concussion^14^. Studies have shown that the brain deformations (strain) have strong dependence on the frequency of the input loading^15^, with shear waves penetrating deeper into the brain at lower frequencies. Recently, Laksari et al. published a cadaver based impact experiment that identifies the peak relative brain motion at around 20 Hz^6^ and also derived the spatiotemporal characteristics of the brain during head impacts using mode decomposition techniques^16^. This involved using dynamic mode decomposition on brains nodal displacements, where it was found that the modal displacement amplitudes and peak strains in the brain have frequencies in the 20–40 Hz range. This relatively wide range is due to the non-homogenity of the brain, since different parts of the brain have different physical properties. This work also uses modal analysis to understand the major difference between head impact cases that lead to loss of consciousness and the ones that did not. The primary interest of this work is to understand the frequency of vibrations set in the human brain upon a blunt impact. To study the frequency of vibrations that are triggered in the brain upon a blunt impact, we use a brain surrogate developed by a team of researchers at Michigan State University^17^. This biofidelic brain system model, also called a phantom, has been used in multiple experiments to study possible injury mechanisms of a TBI. The phantom was first conceptualized by Miller et al. where they performed computational simulations on the model in order to study blast over-pressure correlations to TBI. Their three-dimensional representation consists of a simplistic and idealized model of a human brain as shown in Fig. 1b and c. This model demonstrates the overall size characteristics of the human brain with the interactions of the sulci and gyri (folds and grooves). This phantom was reviewed and verified by a board-certified neurologist who confirmed the phenomenology similarity with a real brain^18^. The computational model was able to show higher strains within the brain interfaces and folds, supporting the hypothesis that blast TBI causes more damage in sulci and gyri^18^. To build a brain phantom suitable for experiments, Wermer et al. studied different materials to determine the best biofidelic representative of the brain matter. Their study included polyacrylamide (PAA), bovine skin/bone, and ballistic gelatin on which they performed mechanical testings of tension, compression, and shear. These mechanical properties were compared with literature values for human and porcine brain tissue. PAA was found preferable to simulate brain tissue due to its multiple material properties and ease fabrication^19^. Utilizing this gelatin and the aforementioned geometry, Kerwin et al. performed an experimental study where the head surrogate was placed in a flexible plate and subjected to a blunt impact and presumed cavitation (creation and collapse of vapor bubbles in the liquid) was observed in between the sulci of the brain. This was the first sight of cavitation in an experiment outside of computational models due to head trauma. This observation was possible due to the gyrated geometry of this phantom, something other experimental models have not been able to replicate^17^. Although the brain phantom used in this work does not fully simulates a real brain with ventricular cavities, lobe differences, and other anatomical factors, its current geometry has made possible experiments with visualization of brain mechanics that contribute to the knowledge of TBI mechanisms.

The focus of this work is to study two different methods to obtain the frequency of vibration upon impact to the brain. One of them uses a flexible ferroelectret nano generator (FENG) as an invasive sensor—which provides an electrical signal in response to applied pressure (or stress)—and the other makes use of particle image tracking to obtain strain. The FENG has been validated in the past as a microphone by Li et al.^20^ and Dsouza et al.^21^ and in pressure sensing applications by Cao et al.^22^, these use cases are similar to the one applied in this work. To validate both PIV and the FENG, the flexible sensor is placed in a brain region similar to the one where the particle images are being tracked. The results from both approaches are normalized and compared in the frequency domain using an Fast Fourier Transform (FFT) to highlight the frequencies of vibration. This information will provide time-dependent information of a wave traveling through the brain, and play a key role in identifying the severity of a given impact, thus helping in the assessment and diagnosis.

## Experimental setup

The setup comprises three major components: (1) a biofidelic test object (hereinafter referred to as the “phantom”), which represents the human brain; (2) a FENG device, which serves as an invasive vibration sensor; and (3) a data acquisition setup used to monitor the FENG device’s electrical signal output upon impact, as well as capturing images of particles embedded in the phantom. Experiments were performed in a custom-built drop tower as shown in Fig. 1a. The system is intended to impact the phantom with a free-falling load (2.5 kg released at 0.5 m from the subject), producing a linear acceleration impact that allows more control over the desired kinematic. A free-falling load results in a significant impact that still allows for multiple tests without damaging the phantom’s structural integrity. Since the initial position of the mass was at rest, conservation of energy calculations were performed to convert that potential energy into kinetic energy upon impact. Theoretical values are shown in Table 1, where friction and drag losses are neglected.

**Table 1:** Impact theoretical conditions.

Characteristic	Symbol	Value	Units
Mass	*m*	2.5	kg
Speed	*v*	3.13	m/s
Momentum	*p*	7.83	kg m/s
Kinetic energy	$E_k$	12.26	J

### Biofidelic brain and neck

The subject utilized in this study consists of the model of a human brain shown in Fig. 1b and c as introduced in the previous section. The cross-sectional view in the axial plane of the brain is extruded to simulate the total brain volume, creating a computer aided design which was utilized to created a mold as shown in Fig. 1d. To simulate the brain tissue, the hydrogel PAA was utilized at a weight concentration of 10%, which represents the white matter of the brain. The subject was created starting with 60 g of Acrylamide (purity $\ge$ 98%, gas chromatography, Sigma-Aldrich, USA) dissolved in 600 ml of deionized (DI) water. Then 2 g of N,N’-methylenebis (MBA,purity 99%, Sigma-Aldrich, USA) was added and let to homogenize while stirring occasionally. Once dissolved, 0.52 g of Ammonium Persulfate (APS, purity $\ge$ 98%, Sigma-Aldrich, USA) was mixed in. The polymerization time was accelerated by adding 0.6 ml of N,N,N’,N’-tetramethylethylenediamine (TEMED, ReagentPlus, 99%, Sigma-Aldrich, USA). This solution was then poured into the mold filling about half to the extrusion width and left to solidify. Once cured, a layer of micron-size sand particles are randomly dispersed for visualization purposes. Finally, another solution of 600 ml was prepared and added on top to obtain the brain tissue model shown in Fig. 1e.

Finally, the PAA brain model is placed inside a 3D-printed poly(lactic acid) (PLA) skull with 50% rectilinear infill which represents the skull bone composition of a spongy bone (diploë) between two layers of compact bone. The sides of this PLA skull are sealed with acrylic windows to allow imaging, and the PAA model is free to move inside the skull filled with DI water to simulate the cerebral spinal fluid (CSF)^23^. The head model was finalized by attaching the PLA skull to a Hybrid-III Neck with an custom built corner as shown in Fig. 1a. Due to the fabrication process, this phantom does not account for individual lobes and strains that might occur between them. In addition, ventricular physiology is not accounted for, a concept that is being considered for future phantom designs. The 3D-printed skull at 50% infill has similar physiological features compared to a human skull by having a porous center enclosed by outer solid shells. However, a single size and volume geometry was utilized—representing the 50th percentile for a biological male human.

### FENG implementation

**Figure 2:**
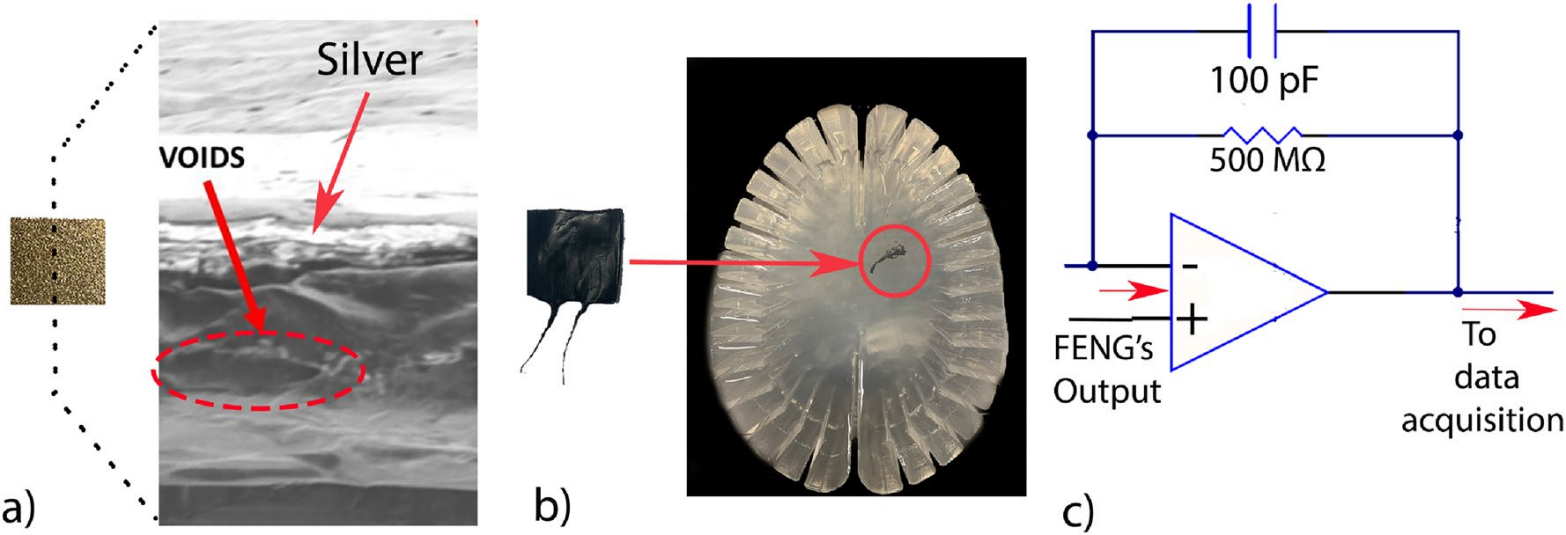
(**a**) FENG after metal electrode deposition and crossection showing the voids and silver electrode, and (**b**) after coated with liquid electrical tape and placed inside the brain phantom. (**c**) Charge mode amplifier circuitry to which the FENG output is fed.

The device consists of FENG, which is formed from a flexible, thin polypropylene (PP) piezoelectret film with micrometer-scale “quasi-dipoles” across its thickness and electrodes at both surfaces as shown in Fig. 2a. Details on the fabrication and operation of these FENG devices can be found elsewhere^24^. Briefly explained, applying a mechanical stress reshapes internal macro-sized dipoles, generating charge accumulation in the electrodes, thus resulting in an electrical output in the form of an electric potential difference between the electrodes, or the flow of charge across a load connected between those electrodes (i.e., voltage or current)^25^. This phenomenon is commonly referred to as “quasi-piezoelectricity”, and these devices have been demonstrated to be useful in a wide variety of applications such as loudspeakers, microphones^20,26^, structural health monitoring^27^ and energy harvesting^28^. The FENG was also used in development of flexible self-powered patch to estimate head rotation kinematics and there-by assist in concussion prediction^29^.The mechanical compliance, overall size, and higher sensitivity compared to other flexible sensors makes FENG devices suitable candidates to measure vibration inside the phantom.

The surface area of the FENG used in this work is 2 cm x 2 cm, with a thickness of $\sim$ 100 μm. The FENG and the electrical connections need to be insulated in order to protect it from the cerebrospinal fluid, which is represented by water in these experiments. This is achieved by coating the FENG with commercially available liquid electrical tape and using enameled copper wire to ensure a very low profile terminals as shown in Fig. 2b. The insulated FENG is then carefully embedded in the PAA gelatin as shown in Fig. 2b, created with the procedure explained in the previous section. This location was chosen based on the research conducted by Okamoto et al.^15^. This study shows that the AP wave (Anterior-Posterior) displacement was the highest in this region as shown in 2b. The terminals are drawn out of the phantom while ensuring that the phantom remains sealed during impact.

## Methods

Two sets of data acquisition hardware were used in this experiment. Electrical signals from the FENG were recorded using a data acquisition system from National Instruments (NI-DAQ 6003) in conjunction with LabVIEW at 50000 SPS (Samples per second). The voltage response from the FENG is first fed to the charge mode amplifier as shown in Fig. 2c and the output of this charge mode amplifier is sent to the NI-DAQ for data recording. This process isolates the system’s voltage response (gain) from parasitic capacitances, such as those created by instrument connections and cables. All the measured data is in time domain, but a Fast Fourier Transform is used for frequency analysis.

**Figure 3:**
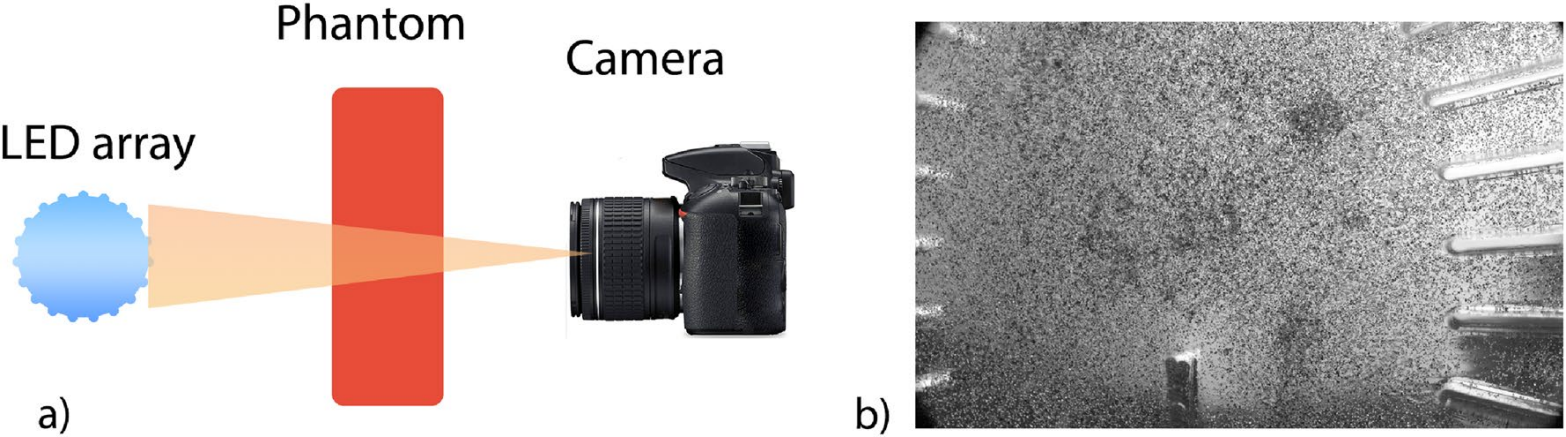
(**a**) Side view of the setup emphasizing on the LED array and camera. (**b**) Sample image that the camera captures. The fine particles are clearly visible in this image.

Images of the biofidelic phantom are captured using an ultra-high-speed camera (Phantom V2512 Series) at 25000 frames per second (fps) with full resolution settings (1280 $\times$ 800 pixels). An array composed of a high-intensity LED (LaVision VI-Strobe LED 120W v2, Göttingen, Germany), concave lens, and a diffusion sheet was utilized to provide back illumination and capture the images for the duration of the impact (Fig. 3a). The camera frame covers an area of approximately 100 mm wide by 60 mm high, which encompasses majority of the phantom as seen in Fig. 3b. The camera image resolution was 0.08 mm/pixel in real-to-machine units. The processing of the images using Particle Image Velocimetry(PIV) are described in the following “Particle image velocimetry implementation” Section.

### Particle Image Velocimetry implementation

**Figure 4:**
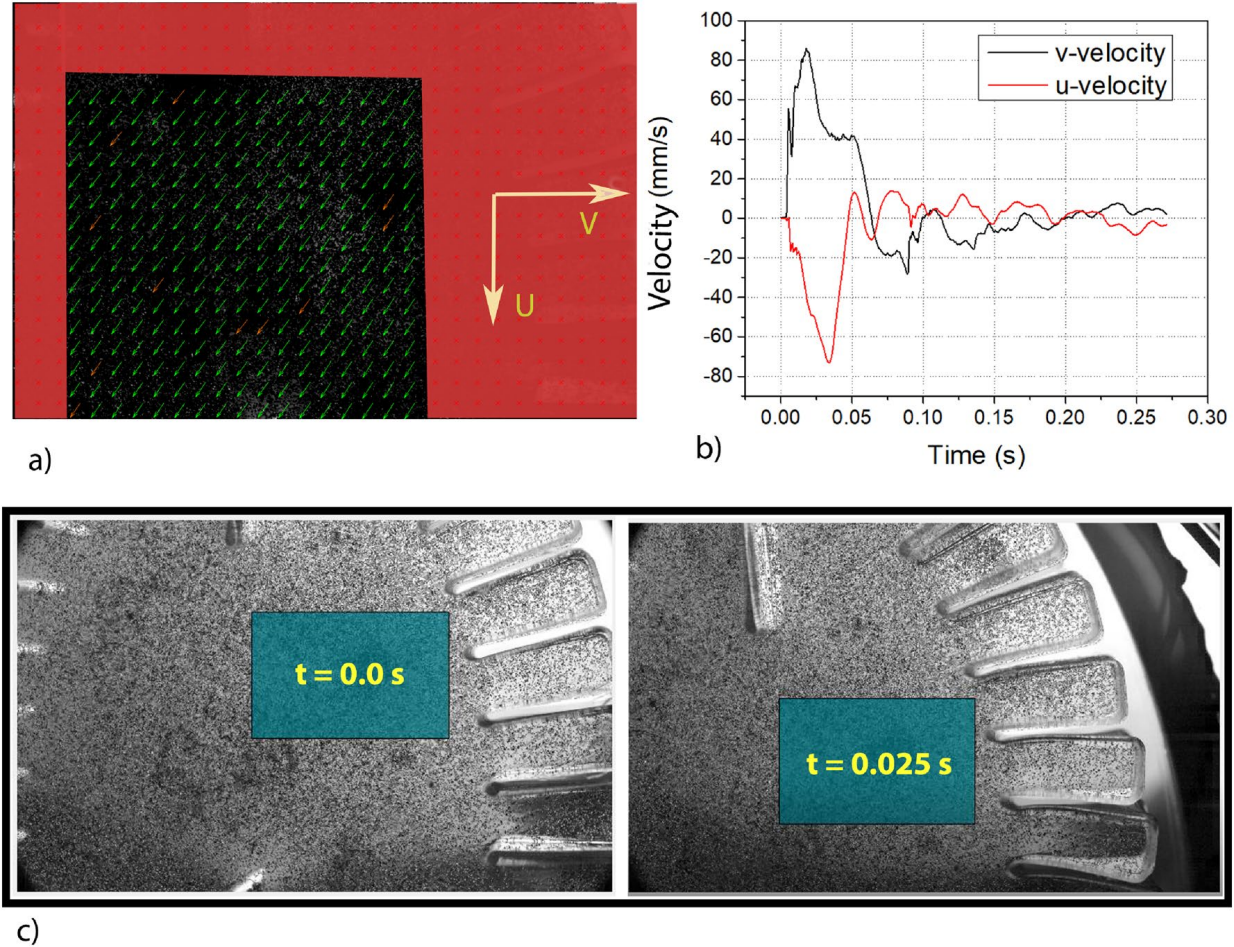
(**a**) Frame of the phantom after impact with displacement vector field overlaid upon it depicted by green arrows. (**b**) Instantaneous average bulk velocity of the phantom decomposed into *u* and *v* components. (**c**) Region of interest (shaded in blue) tracks the bulk motion.

PIV is typically used to calculate the velocity field in fluid flows. To this end, PIV uses a FFT-based algorithm to calculate the cross-correlation between corresponding regions of consecutive images^30,31^. This algorithm is applied herein to images of tracer particles embedded in a viscoelastic material^32^. After normalizing by the grid size, this velocity field is a direct measurement of the instantaneous strain-rate field. The frame rate used for this measurement of the strain-rate field was 2500 frames per second. This frame rate was chosen so as to faithfully resolve parameters of interest for a given interrogation window. The PIV analysis was performed in the same general region of interest (ROI) in which the FENG was embedded. This ROI is demarcated by the blue squared region in Fig. 4; and the time inside the box represents the time elapsed since dropping the brain phantom. Everything outside of the ROI was eliminated from the figure with a binary mask.

The tool was applied to an initial interrogation window of 256 $\times$ 256 pixel, then decreasing by 50% until reaching a 16 pixel $\times$ 16 pixel window. Although an initial interrogation window is typically smaller, the chosen size allowed tracking of particles even when the surrogate head displaced significantly. Spurious vectors within the region of interest were filtered out using image based validation which considers features such as contrast within a given region, and velocity-based validation where outstanding erroneous velocity vectors can be ignored. The strain-rate between consecutive frames was extracted from the PIVLab application and imported to a database for computational analysis (see method 1 in the supplementary material).

Since the head surrogate is fixed to a flexible mechanic neck, it is free to move out of its initial position in the camera’s field of view. To address this, an in-house code was used to track the local deformations of a specific region in the brain. The chosen region of interest moves every frame according to the mean of the *u* and *v* velocity components, and is further explained in the “Results” Section. This information was used to calculate the average strain-rate within this region of interest.

## Results

In order to obtain the strain using PIV analysis, it is vital to track the bulk motion of the phantom so as to obtain the strain from a constant region of interest.

**Figure 5:**
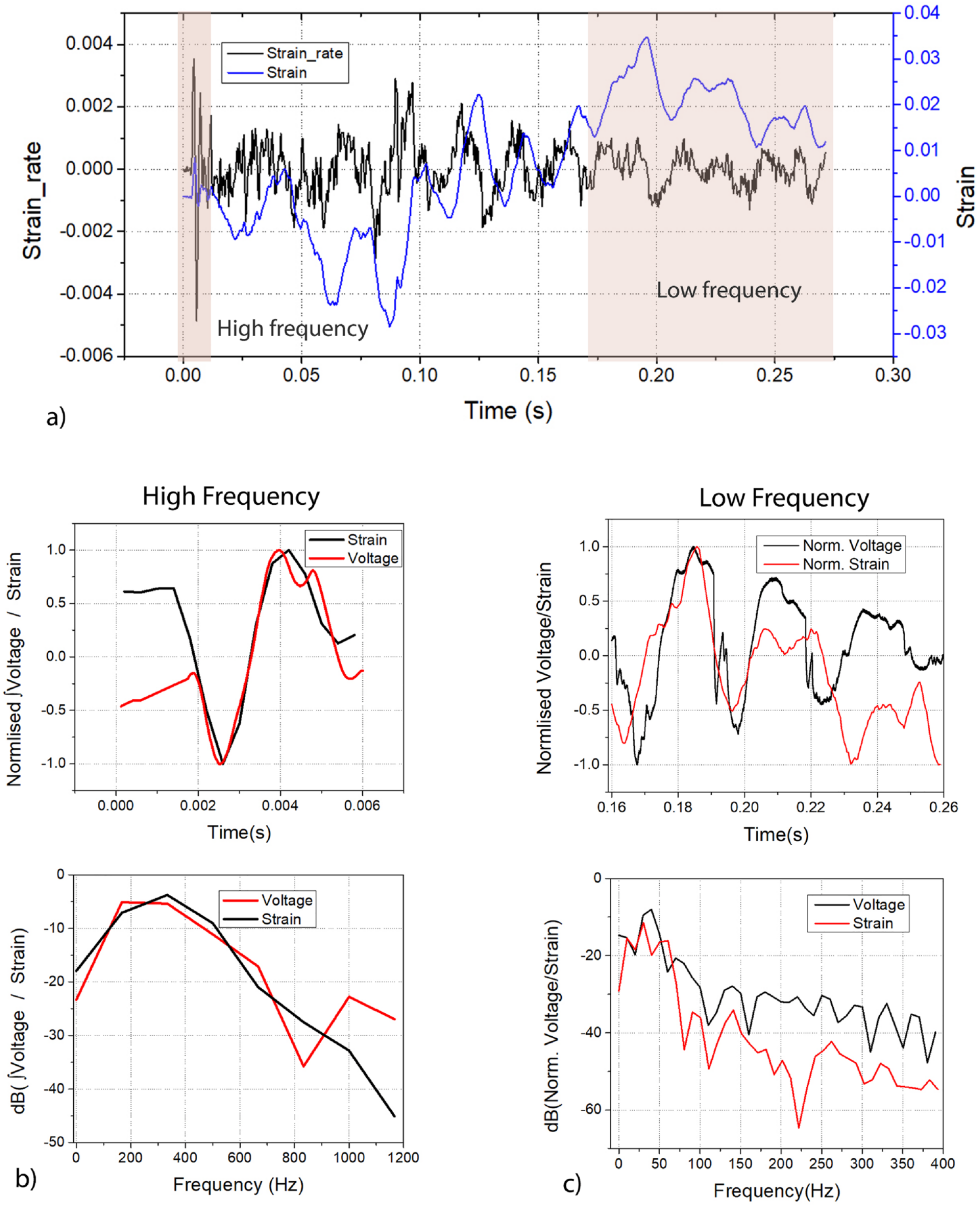
(**a**) Strain and strain rate derived from PIV analysis. (**b**) (top) Strain obtained from PIV and the integral of voltage from the FENG. (0–6 ms) bottom) FFT of strain obtained from PIV and the integral of voltage from the FENG. (**c**) (top) Normalized strain and voltage response from the FENG between 160–260 ms after impact. bot) FFT of normalized strain and voltage.

This is performed with the help of average “v- velocity” and “u- velocity” obtained across the entire computational area as shown by the black region in Fig. 4a (the red shaded region is neglected). These velocities are shown in Fig. 4b. Figure 4c shows the captured frames from different instances in time with blue region overlaid highlighting the tracking of the bulk motion using average velocities. The strain rate from this region of interest is obtained across every frame and is shown in Fig. 5a, along with strain, which is calculated from the strain rate. This strain is later used to analyze the modality of the deformations. The low frequency and high frequency time intervals are highlighted in the same graph.

**Figure 6:**
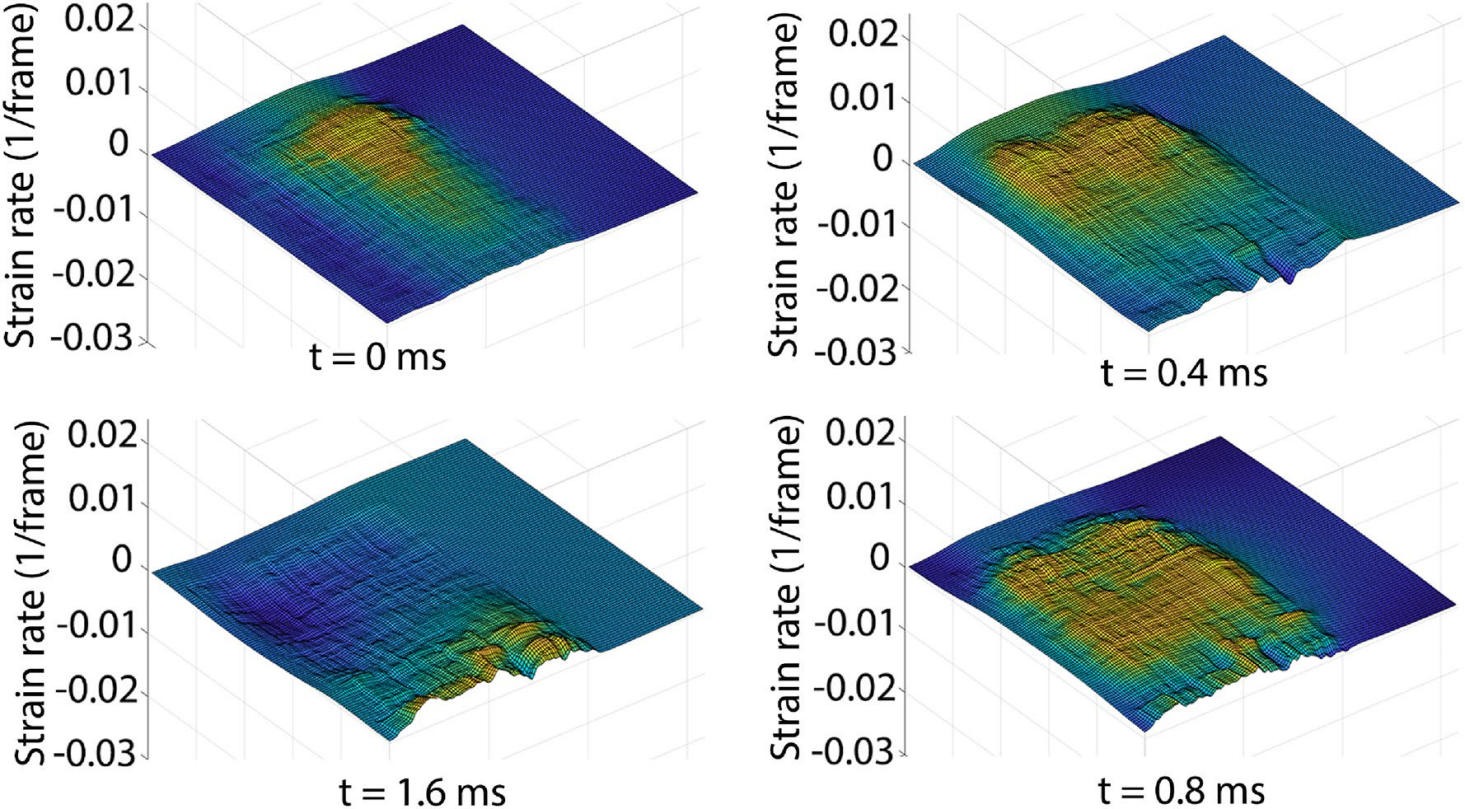
Four frames and their corresponding surface plot to interpret the progression of the shock wave. t = 0 depicts the moment of impact and progressing clockwise.

In order to validate the results from the PIV, we observe the strains generated due to the shock-wave that propagates upon impact. This is shown in Fig. 6. The progression of strain on the brain tissue can be found in supplementary videos 2 and 3.

The visco-elastic nature of the brain brings a non-linear, frequency-dependent, relationship between stress and strain^33^. The FENG device provides voltage signals in response to stress, while the PIV analysis captures strain. Thus the study of results from both these approaches will provide useful insights into the non-linear stress-strain relationship of the brain. A Kelvin-Voigt visco-elastic material can be electrically modeled as a constant current source in series with parallel resistor-capacitor (RC) circuit^34^. In this analogy, the current source represents the applied stress and the voltage across the capacitor represents the strain.

When the stress changes slowly i.e. input of relative low frequency, the strain follows the stress. This is similar to how the voltage across a capacitor in an RC circuit is in-phase with the input. However, when the stress changes at a higher rate, the strain lags, which again follows the behaviour of an RC circuit. This can be also approximated as an integrator circuit, where a square wave input to the RC circuit produces a triangular wave output. The high frequency components of the traveling wave occur during the initial phase of the impact, when the brain behaves predominantly as a viscous medium. This is supported by the positive correlation of the time-domain and FFT of the strain obtained from PIV and the numerical integration of FENG’s response as shown in figure 5b. The FFT’s correlate by 0.795 (Pearson’s R). Similarly, the low frequency domain can be observed after $\sim 160ms$ from impact when the brain begins to oscillate around its natural frequency. The normalized voltage and strain after this time frame, and the corresponding FFTs are shown in Fig. 5c. In both frequency spectra, we can see that the dominant frequencies are between 15 Hz and 60 Hz, with a peak around 25 Hz. There are other higher frequencies present in the voltage response of the FENG which could be generated from the gyri’s local oscillations. Nevertheless, the spectra have a correlation of 0.82 (Pearson’s R). This supports that the brain is exhibiting a more elastic behaviour.

This high positive correlation supports that the FENG, although invasive, can be used in studying frequencies of brain vibrations under any form of blunt impact. The FENG involves minor signal conditioning circuitry and data acquisition hardware to capture the several metrics around the impact studies. The PIV is non-invasive, but the setup is more complex, involving more equipment (e.g. high speed camera) and being more susceptible to testing parameters (e.g. illumination). Also, it relies on the subject having minimal displacement so that it does not gets outside the frame boundaries; a situation that occurs mainly with larger impact magnitudes.

## Conclusion

The area of understanding traumatic brain injuries has been forever evolving. Several hypothesis have been proposed thus far with the help of cadaver based studies, finite element simulations and biofidelic brain substitutes with every experiment leading to an improvement in defining markers to reliably identify the type and extent of brain injury. Along the same line, this work presents two methods for understanding the modality of brain vibrations that are set in a brain phantom when subjected to a blunt impact. PIV, being a non-invasive but bulkier in setup and the FENG, an invasive yet simple setup show great potential for studies of this nature. These proposed methods can be employed to characterize an array of blunt impacts across varying intensities, locations and angles of impact with respect to the modality of deformations that are excited in the phantom. Although the current phantom does not account for the diverse brain physiology the future versions will accommodate individual lobe-specific properties. These results in conjunction with existing theories will further the very understanding behind traumatic brain injuries.

## Supplementary Information

Supplementary Information 1.

Supplementary Information 2.

Supplementary Information 3.

Supplementary Information 4.

Supplementary Information 5.

## Data Availability

The datasets used and/or analysed during the current study are available in the excel file included in the supplementary material section.
