# Supplementary material for: Measuring vibrations on a biofidelic brain using ferroelectret nanogenerator
Source: Sci Rep. 2023 Jun 2;13:8975. doi: 10.1038/s41598-023-35782-5 (PMC10238378; doi:10.1038/s41598-023-35782-5)
Supplement: Supplementary file 5 — Supplementary Information 5. [file 41598_2023_35782_MOESM5_ESM.docx]

# Supplementary method 1

This supplementary method highlights the setting used in PIVLab to help reader to recreate the analysis.


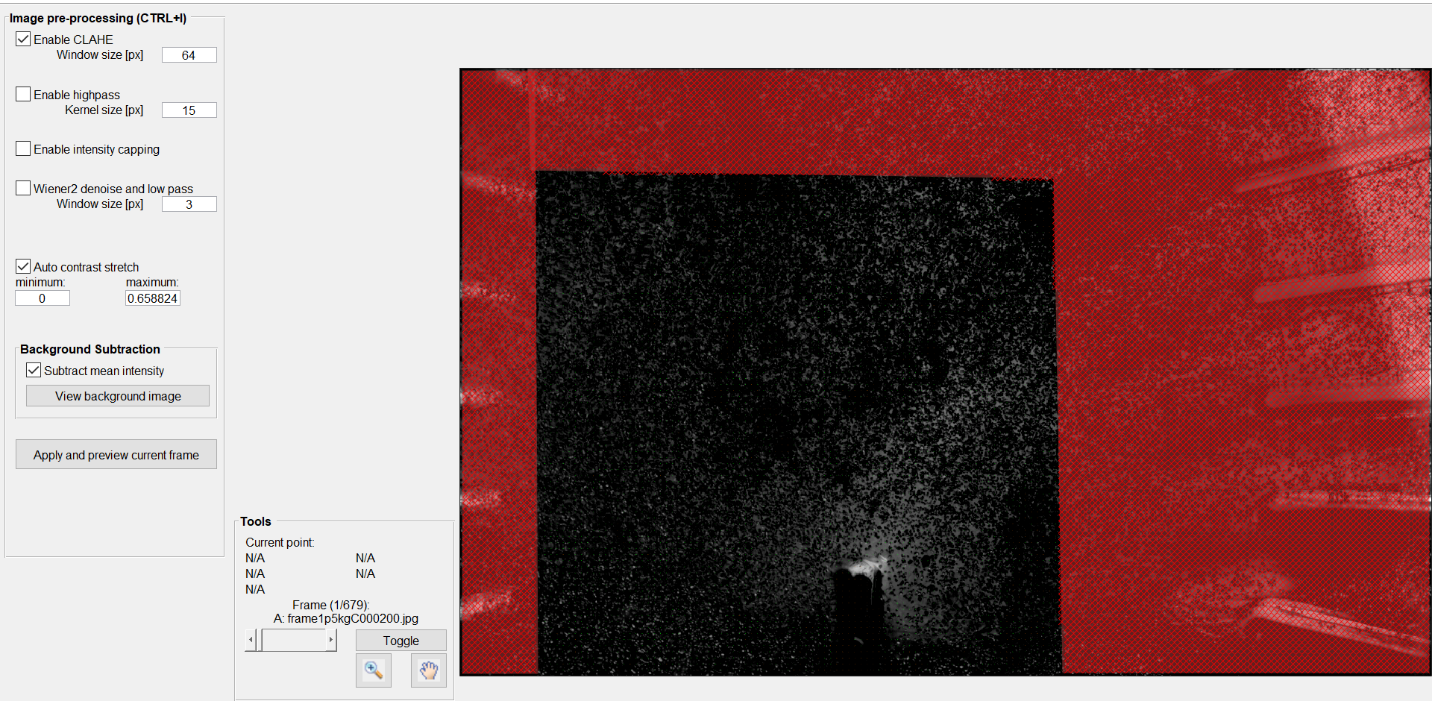


Supplementary Figure 1 : Image pre-processing tab in PIV lab. The red area is a mask to exclude the area under it from computation.

Please note that “subtract mean intensity “ option is only available with “Timewise” analysis, i.e. only when subsequent images are used as references and not when all the frames are referenced to the first frame. The authors chose to use timewise – analysis because of the large bulk movement of the phantom.


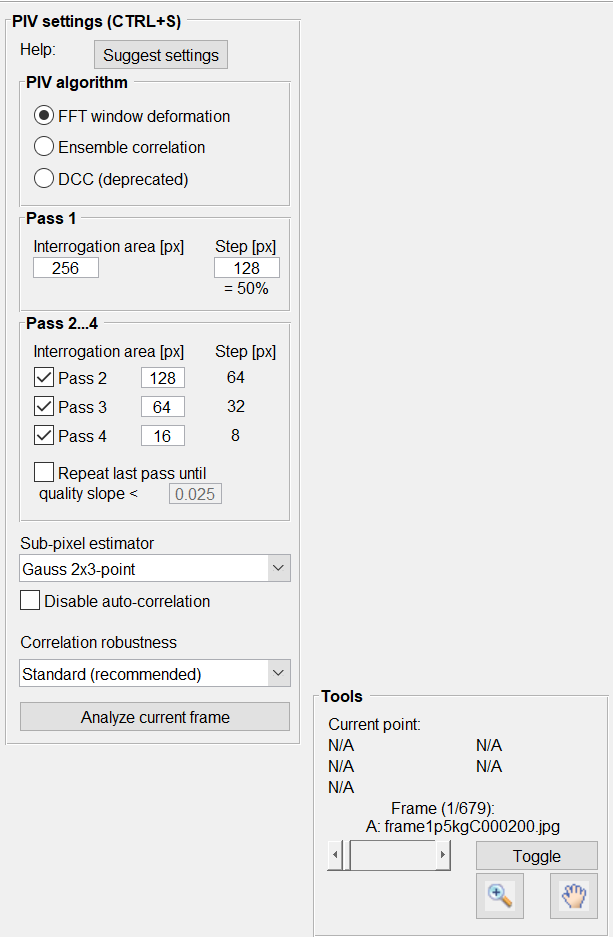


Supplementary Figure 2: PIV setting used for analysis.


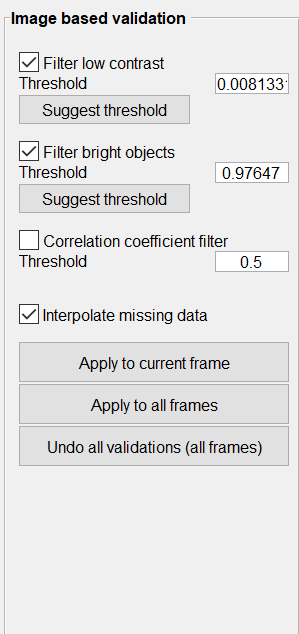


Supplementary Figure 3: Setting on image based validation tab.

# Supplementary movie 1

This video was extracted from the PIVLab tool on Matlab software. This is how each frame is being processed for determining the vectors denoted by green arrows that show the displacement of several sections across the entire frame. The area under the red mask is neglected from computation.

# Supplementary movie 2

Strain of the entire area included in the computation as a surface plot along with the raw frames captured. This video only focuses on the shock wave moving through the brain.

# Supplementary movie 3

Strain and the region of interest highlighted over the phantom. This also shows how the region nearly tracks the bulk motion.
